# Abundant Intracellular IgG in Enterocytes and Endoderm Lacking FcRn

**DOI:** 10.1371/journal.pone.0070863

**Published:** 2013-07-29

**Authors:** Sudhasri Mohanty, Jonghan Kim, Latha P. Ganesan, Gary S. Phillips, John M. Robinson, Clark L. Anderson

**Affiliations:** 1 Departments of Internal Medicine, The Ohio State University, Columbus, Ohio, United States of America; 2 Center for Biostatistics, The Ohio State University, Columbus, Ohio, United States of America; 3 Department of Physiology and Cell Biology, The Ohio State University, Columbus, Ohio, United States of America; 4 Department of Pharmaceutical Sciences, Northeastern University, Boston, Massachusetts, United States of America; McGill University, Canada

## Abstract

FcRn, a non-classical MHCI molecule, transports IgG from mother to young and regulates the rate of IgG degradation throughout life. Brambell proposed a mechanism that unified these two functions, saying that IgG was pinocytosed nonspecifically by the cell into an FcRn-expressing endosome, where, at low pH, it bound to FcRn and was exocytosed. This theory was immediately challenged by claims that FcRn specificity for ligand could be conferred at the cell surface in neonatal jejunum. Assessing Brambell's hypothesis we found abundant nonspecifically endocytosed IgG present in the cytoplasm of FcRn^−/−^ enterocytes. Further, IgG was present in the intercellular clefts and the cores of FcRn^+/+^ but not FcRn^−/−^ jejunum. FcRn specificity for ligand could be determined within the cell.

## Introduction

The father of maternal-fetal IgG transport, Rogers Brambell, hypothesized that a single Fc receptor was responsible remarkably for two widely disparate critical bodily functions; for the transport of IgG across fetal or neonatal tissue barriers and for the regulation of the rate of IgG degradation throughout the life span of the individual [1], [2]. These two functions took place in entirely different organs during entirely different developmental periods; transport in the transient placenta or yolk sac (YS) or neonatal gut, regulation of degradation at unspecified sites in the long-lived body. His model, accommodating all observations of the time, predicted that the single receptor worked at both sites in the same fashion. It encountered and bound nonspecifically-pinocytosed IgG in an intracellular vesicle and ferried it back out of the cell, effectively separating bound IgG from excess IgG and all other plasma proteins. Thus the receptor served as an effective ‘transporter’ by moving IgG across the cell, and it regulated the rate of IgG degradation by ‘protecting’ IgG from the lysosomal degradation pathway. Brambell's hypothetical receptor was eventually shown to be FcRn, a non-classical MHCI molecule, that bound IgG at the low pH of acidic endosomes but showed no attraction for IgG at physiologic pH [3]–[7]. FcRn, thus, fulfilled the need for a receptor that would function intracellularly. It was found also to bind and protect albumin from degradation in a like manner, explaining thus many old observations about albumin turnover [8]–[10].

One of the essential features of Brambell's initial hypothesis held that specificity of the receptor for IgG was dictated intracellularly and not at the plasma membrane. Immediately, this important feature was challenged. Rodewald and Waldmann, independently, claimed that in the neonatal gut, the receptor conferred specificity for ligand at the enterocyte plasma membrane [11]–[14]. Shortcomings of this view were apparent. Some suggested alternative interpretations of the published data; others countered by showing that the pH of the luminal contents did not affect the rate of IgG transport across the gut [15], still others noted that postulating specificity of the receptor at two different cellular sites defied the principle of parsimony. Further, Rodewald, using an anti-FcRn mab, eventually modified his earlier conclusion, observing that the ‘vast majority’ of enterocyte FcRn was intracellular and not on the plasma membrane [16]. Nevertheless, the view that FcRn conferred its specificity at the plasma membrane of the enterocyte has persisted, catalyzing considerable study of surface FcRn-mediated endocytosis of IgG [17]–[30].

Where in the enterocyte FcRn first manifests its specificity for ligand, either at the cell surface or within the cell, is a controversial yet crucial issue: It is crucial because IgG in the gut lumen might theoretically move through the enterocyte by two pathways: Either it could bind at low pH to a small number of surface-expressed FcRn and be pinocytosed into an intracellular compartment (a). Or, it could be nonspecifically pinocytosed by the enterocyte and move to acidic endosomes expressing FcRn (b) where it meets and binds FcRn. Whether these two intracellular compartments (a and b) are the same, how they might interact, by what pathways might they transit the cell, are important mysteries that can only be resolved by additional experimental work. It seems eminently possible that IgG from the two compartments (a and b) moves within the cell along independent pathways, in which case defining these two compartments remains crucial. The pathways cannot be assumed to be identical.

We have addressed this 40 year-old controversy by contributing additional data. The availability of a mouse strain lacking FcRn [31] gave us the opportunity to test a prediction that might support the Brambell hypothesis. The prediction is that if ligand-specificity were conferred intracellularly, then FcRn^−/−^ enterocyte cytoplasm should show abundant nonspecifically pinocytosed IgG. Using the new hamster anti-mFcRn antibody [32] and confocal immunofluorescence (IF) microscopy with quantitative imaging, we assessed the cellular localization of FcRn and IgG in both gut and YS of FcRn^−/−^ and FcRn^+/+^ mouse strains. We found that the nonspecific uptake of IgG by the enterocyte is high, compatible with intracellular determination of specificity. Further, we confirm and extend our prior description of abundant intracellular IgG in the endoderm lacking FcRn [33]. Brambell's hypothesis continues to survive.

## Materials and Methods

### Reagents

Armenian hamster anti-mouse FcRn serum and pre-immune serum were provided by Dr. A. Shaw (Washington University, St. Louis, MO) [32]. A rabbit anti-rat FcRn antiserum was a gift from Dr. P. Bjorkman (California Institute of Technology, Pasadena, CA). The Cy3 dye-conjugated goat IgG anti-Armenian hamster IgG and DyLight 488 dye-conjugated polyclonal goat IgG anti-mouse IgG heavy chain Fc fragment were obtained from Jackson ImmunoResearch, and HRP-conjugated goat IgG anti-rabbit IgG was obtained from SantaCruz Biotechnology. Alexa 633 dye-conjugated phalloidin and ProLong anti-fade mounting medium were obtained from Invitrogen. DAPI was purchased from Sigma–Aldrich and SuperfrostPlus slides from Fisher Scientific.

### Animals and breeding

Breeders of the FcRn alpha chain-knockout strain (B6.129X1/SvJ*Fcgrt^Tm1Dcr^*; FcRn^−/−^) and its wild-type strain (C57BL/6J; FcRn^+/+^) were obtained from Dr. D. C. Roopenian of The Jackson Laboratory (Bar Harbor, ME) [31]. Mice were bred in house to obtain wild-type (FcRn^+/+^) and knockout (FcRn^−/−^) strains from heterozygous mothers as described earlier [33]. All animal studies were approved by The Ohio State University Institutional Animal Care and Use Committee.

### Harvesting neonatal intestine and term fetuses

Neonatal intestines and term-conceptuses were harvested as described previously [33], [34]. Briefly, the small intestines of isoflurane anesthetized 10 day-old pups of heterozygous mothers were resected en bloc by cutting at the pylorus where the stomach joins the small intestine and at the end of the ileum where the caecum continues. Within the 15–18 cm small intestine, three contiguous segments could imprecisely be discerned; the 1 cm C-shaped duodenum following the stomach and connected to the diaphragm by the ligament of Treitz; the milk-filled pinkish jejunum continuing to mid-small intestine; and the pale yellowish ilium containing occasional fecal material. The proximal half of the small intestine was quickly cut into 7 pieces, each one cm long, from the pylorus distalward, including the duodenum and jejunum. For the immunoblot (IB) assay, these 7 pieces were rinsed in PBS, blotted on Kimwipes, quickly frozen, and then stored at −80°C until further use. For the IF assay, each cm long piece was fixed in freshly made 4% paraformaldehyde (w/v) solution. Pup tail clips and maternal livers were used for genotyping. After genotyping, tissues belonging to FcRn^+/+^ or FcRn^−/−^ strains were processed for IB and IF assays.

For YS harvesting, at the gestational age of day 19–20, pregnant female mice were anesthetized under isoflurane and subjected to Cesarean section to obtain near-term conceptuses. Fetal tail tips and maternal livers were obtained for genotyping. The placenta/YS units were fixed in freshly prepared 4% paraformaldehyde in PBS for 2 h at room temperature. The tissue was then washed extensively in PBS, infiltrated in 20% sucrose (PBS) overnight at 4°C, embedded in tissue freezing medium, and frozen at −80°C until further use. The intestine pieces isolated for IF assay were processed in a similar manner.

### Immunoblot analysis

To make tissue lysates for IB, intestine pieces were thawed, homogenized in 60 mM octylglucoside buffer pH 7.4 and processed as described earlier [35]. Protein concentrations were determined by a modified Lowry method (Bio-Rad). Equal amounts of proteins from lysates representing cm 1 through cm 7 tissues from FcRn^+/+^ and FcRn^−/−^ strains were then resolved on 8–16% precast gradient gels (Bio-Rad) and transferred to nitrocellulose membranes (Hybond ECL; Amersham Biosciences). The membranes were blocked in 5% nonfat milk at room temperature for 1 h and probed with rabbit IgG anti-rat FcRn protein and relevant control antibodies overnight on a rocker at 4°C. Membranes were washed and incubated in peroxidase-conjugated secondary antibodies for 1 h at room temperature. After several washes, membranes were subjected to ECL. For quantification, blots were scanned and FcRn signals were analyzed by NIH image J software. Average band densities and standard deviations of FcRn signals for each strain were plotted for all intestinal pieces (cm 1–7). *P* values were calculated using Student's *t* test.

### Immunofluorescence localization of FcRn and IgG in gut and YS

We selected cm 1, 4 and 5 for IF analysis because cm 4 and 5 showed highest and 1 showed lowest levels of FcRn expression in IB assays. Sections of neonatal intestine tissues from FcRn^+/+^ and FcRn^−/−^ strains were cut at 5 µm thickness in a Shandon cryostat (Global Medical Instrumentation) and were collected on SuperfrostPlus slides. The sections were hydrated, blocked in 5% nonfat dry milk, and incubated overnight with 1/1000 hamster anti-mouse FcRn serum plus 1/100 goat anti-mouse IgG heavy chain Fc fragment in blocking buffer at 4°C. Antiserum binding was localized indirectly by Cy3-labeled goat IgG anti-hamster IgG secondary antibodies at RT. Alexa 633 dye-conjugated phalloidin at 1/20 dilution was used in all reactions to mark cell boundaries. Nuclei were stained with DAPI for 10 min and the sections were mounted under cover slips in ProLong mounting medium. Controls included sections incubated with hamster pre-immune serum plus secondary antibodies as well as hamster anti-mouse IgG secondary antibody alone. The control for IgG labeling included DyLight 488 dye-conjugated goat anti-chicken IgY and goat anti-rat IgG antibodies. For detailed analysis and quantification we utilized images of FcRn^+/+^ and FcRn^−/−^ strains from cm 5 tissues, judging that cm 4 and cm 5 tissues gave comparable results. YS sections from FcRn^+/+^ and FcRn^−/−^ strains were processed for IF assays similarly with one modification; *viz.*, Armenian hamster anti-mouse FcRn serum was used at 1/500 dilutions for labeling YS tissue due to lack of strong signal at 1/1000 dilution. Fluorescence and differential interference contrast (DIC) images were collected with a FluoView 1000 Olympus microscope and analyzed with MetaMorph image analysis system (Universal Imaging/Molecular Devices). All images were collected within the linear response range of the camera. We used DIC images to assure correct morphologic identification of tissues [14], [36]–[38].

### Quantitation method

To analyze data objectively and confirm our visual impressions, we employed quantitative methods to compare FcRn and IgG signals in neonatal intestine and YS tissues. For all quantitative analyses we collected the average pixel intensity (API) that is defined as API = X/N, where X is total intensity of all positive pixels above threshold in a given region of interest (ROI) and N is number of positive pixels above threshold for a given ROI. Mean and standard deviation from 300 API were obtained for each analysis.

**Figure 1:**
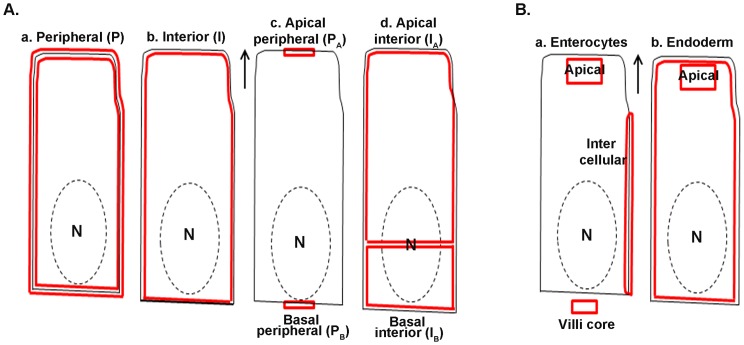
Cartoon of cells illustrating how regions of interest were drawn. Solid black lines represent cell margins. Dashed black lines represent nucleus (N) border. Red lines denote regions of interest, either within or between the red lines. Arrows point toward lumen. Schemes for quantifying both FcRn (**A**) and IgG (**B**) are shown. Details are in text, in Quantitation Method of the Materials and Methods.

To compare the spatial distribution of FcRn expression between endoderm and enterocytes tissue sections were labeled as described in method section. Fluorescence images were collected with an Olympus confocal microscope using FluoView1000 software and analyzed with MetaMorph software. We define peripheral (P) FcRn cellular fluorescence as that coincident with the phalloidin signal, and we measure P as an ROI circumscribing manually the phalloidin signal. We define the internal (I) FcRn cellular fluorescence by circumscribing an ROI within the intracellular edge of the phalloidin signal (Fig. 1Aa and 1Ab). These ROIs were transferred to an identical location in the image showing only the FcRn signal. Thresholds were applied to FcRn images based on non-specific signals, and pixel intensities of ROIs were obtained by the software. This was repeated for 300 YS endoderm cells (n = 3 animals) and 300 gut enterocytes (n = 3 animals), and P/I ratios were calculated for each tissue.

For apical periphery/basal periphery (P_A_/P_B_) calculations a ROI was drawn at the apical and basal margin in phalloidin image to capture coincident FcRn signal (Fig. 1Ac). The ROI was transferred to the FcRn image, a threshold was applied, and pixel intensities were calculated just as was done for the P/I ratio. For apical internal/basal internal (I_A_/I_B_) ratio determination, two ROIs were drawn inside the phalloidin marked cell boundary, an apical ROI above the nuclear midline and a basal ROI below a nuclear midline (Fig. 1Ad). These ROIs were drawn to capture FcRn signals both above and below the nucleus in Golgi complex/trans-Golgi network located differently in YS (below the nucleus) and gut cells (above the nucleus). The ROIs were transferred and pixel intensities were determined after applying thresholds as described above. Various ratios stating differential FcRn distribution between gut and YS cells are shown in Table 1.

**Table 1:** Mean (±1 standard deviation) ratios illustrating differential expression of FcRn between YS endoderm and gut enterocyte cells.

Ratios	Endoderm (n = 3)	Enterocytes (n = 3)	*P* value
P/I	2.0±0.3	1.0±0.0	0.001
P_A_/P_B_	1.0±0.2	2.6±0.7	0.006
I_A_/I_B_	0.9±0.1	1.8±0.3	0.002

P, peripheral; I, internal; P_A,_ apical periphery; P_B,_ basal periphery; I_A_, apical internal and I_B_, basal internal.

To compare endogenous IgG distribution between FcRn^+/+^ and FcRn^−/−^ strains, gut cm 5 sections were labeled for IF as described above. By visually inspecting multiple images from various animals we found three sites where IgG expression and distribution is remarkably different between FcRn^+/+^ and FcRn^−/−^ strains. These sites, chosen for quantitative analysis, are the apical region in enterocytes, the intercellular regions of enterocytes, and the cores of the intestinal villi. Studying the apical region in enterocytes, we drew a rectangular ROI at the apical portion of enterocytes in the phalloidin image and transferred it to the IgG image; threshold was applied and pixel intensity was calculated (Fig. 1Ba). The data were collected from 100 enterocytes from each mouse using a total of 3 mice for each strain. To compare IgG in intercellular areas an ROI was drawn using phalloidin as a marker for the intercellular area. To calculate IgG in villi core a rectangular ROI was drawn below the enterocyte in the core area. The transfer of ROIs, threshold application, and pixel intensity collection were done as described above. For all measurements a total of 300 data were collected for each strain (n = 3 animals). Average intensity and standard deviations are plotted for each area comparing IgG distribution between FcRn^+/+^ and FcRn^−/−^ strains.

We analyzed endogenous IgG distribution in endoderm from FcRn^+/+^ and FcRn^−/−^ strains using a polyclonal goat IgG anti-mouse IgG heavy chain Fc specific antibody and confocal images at high magnification to ascertain whether IgG concentration and location in endoderm are statistically significantly different between the two strains. Our earlier attempt to quantify apical IgG between FcRn^+/+^ and FcRn^−/−^ strains using a less refined approach showed no statistically significant difference. We chose to compare both apical and total intracellular IgG concentrations between these two strains. For this we drew two ROIs as illustrated in Fig. 1Bb. For the whole cell cytoplasm we drew an ROI along the medial aspect of the phalloidin image; for the cytoplasmic area in the apical area alone we drew a box-shaped ROI medial to the phalloidin image in the apical region of the cell. We transferred these two ROIs to the IgG images and applied threshold, as above. The pixels from 300 ROIs for each strain were collected (n = 3 animals). Average intensities and standard deviations are plotted for each area differentiating IgG distribution between FcRn^+/+^ and FcRn^−/−^ strains.

We compared the area occupied by IgG in endoderm of the two strains. For this the IgG images with ROIs drawn to quantify either intracellular or apical areas for average intensity were used. These images were then converted to a binary image by giving a value of 1 to the pixels at and above the threshold and a value of zero to all pixels below threshold. Pixel intensity was not considered. The percent pixel number for each ROI was obtained. The means and standard deviations from 300 ROI of both sorts were plotted. It should be noted that we present no standard curves for IgG or FcRn concentrations, so our comparisons should be understood as comparisons between fluorescent image intensities and not relative protein concentrations. Likewise, we have not quantified the lower limit of IgG or FcRn detection.

### Statistical analyses

Observations equal to zero were omitted for statistical analysis as they were less than 3 times the inter-quartile range below the 25^th^ percentile and it was decided to analyze only detectable signals. This eliminated 9 observations out of the 600 collected (300 per group). For clarity in the rest of the manuscript we still label these groups as ‘300’ even though a few observations have been eliminated. To estimate the intraclass correlation coefficient to determine within-mouse and between-mouse variance we used a component variance analysis. The intraclass correlation rho (ρ) is defined as the ratio of the between-mouse variance to the total variance. Values close to 1 indicate that most of the variability is between the different mice while values near zero indicate that most of the variability is within the mouse. The average pixel intensity was natural log-transformed to meet the normality and homoscedasticity assumptions necessary for regression analyses. As the observations are technical replicates the transformed data were averaged over the three mice before analyzing by linear regression to test if groups were different. The groups were defined by the P/I, P_A_/P_B_, or I_A_/I_B_ ratios for the endoderm *vs*. enterocyte FcRn distribution analysis; or the gut IgG levels in FcRn^+/+^ *vs.* FcRn^−/−^ strains in the apical, intercellular, and core regions of the neonatal gut cell; or the endoderm IgG levels in FcRn^+/+^ *vs.* FcRn^−/−^ strains in the apical region and the whole cell. The analyses used software Stata 12.0 (Stata Corporation, College Station, Texas).

### Intra- and inter-animal variations

Intraclass correlation coefficient ρ values were calculated to assess the intra- and inter-animal variations. We found that all ρ values were near zero indicating that most of the variability was within the animals and that variation among the animals was minimal. Thus the number of animals employed for each analysis is sufficient as the addition of more animals would not significantly increase the power to detect differences.

## Results

### FcRn expression along the length of neonatal intestine

**Figure 2:**
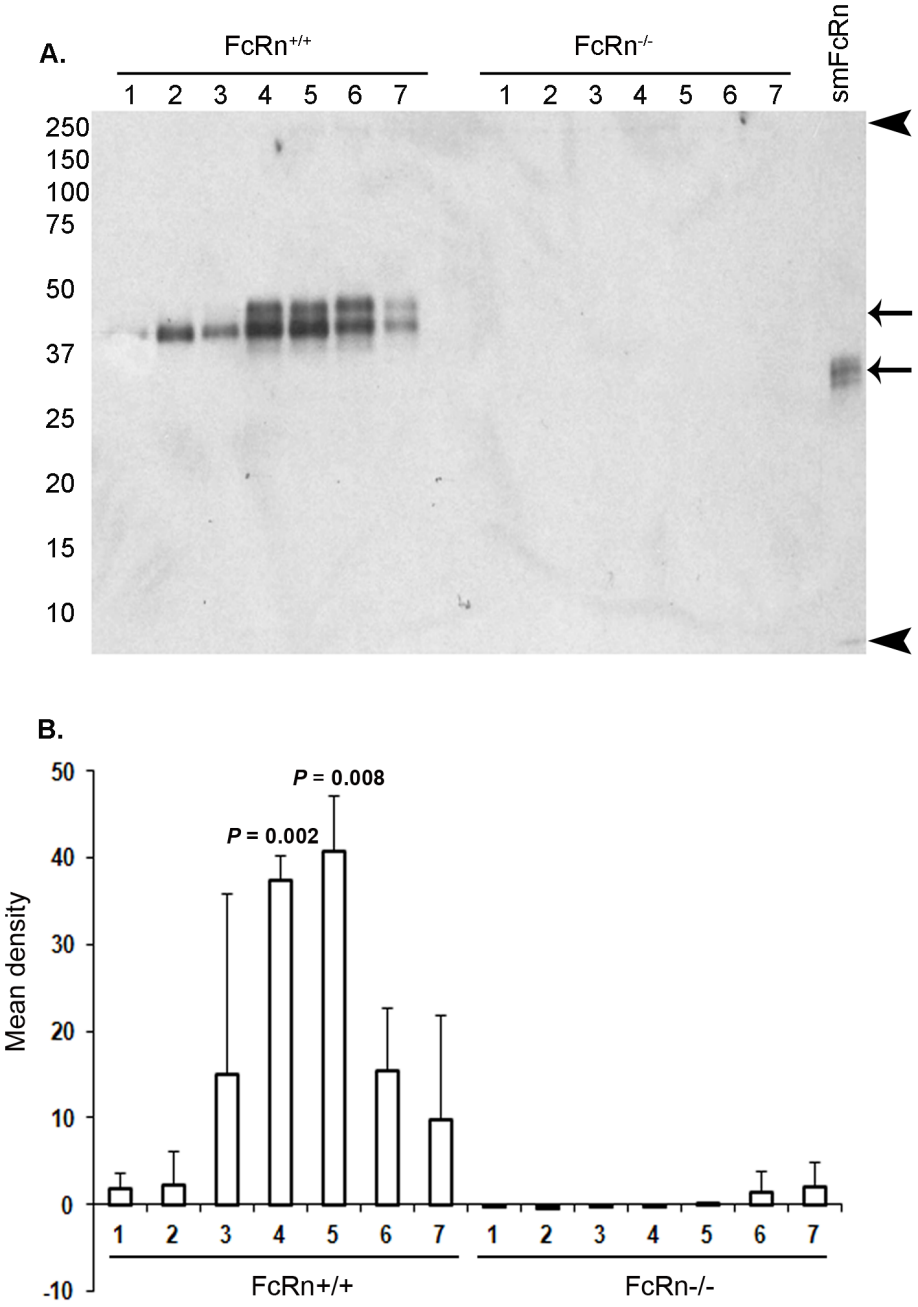
FcRn expression along the linear length of neonatal intestine. **A.** An immunoblot using rabbit anti-rat FcRn antibody shows FcRn expression along a 7 cm length of neonatal intestine cut into 1 cm pieces from pylorus through duodenum and into jejunum, comparing tissue lysates from FcRn^+/+^ and FcRn^−/−^ strains. Numbers at left margin shown are MW markers in kDa. The top arrow at right margin indicates two prominent native FcRn proteins in gut, and the bottom arrow indicates recombinant FcRn protein, soluble mouse (sm) FcRn. Arrowheads mark top and bottom of the gel. Figure is representative of 3 immunoblots. **B.** Bar graph expressing the means and standard deviations of immunoblot-derived band densities for FcRn expression in cm 1 thru cm 7 intestinal pieces from FcRn^+/+^ and FcRn^−/−^ strain tissues (n = 3 FcRn^+/+^ and 3 FcRn^−/−^ mice). The *P* values shown on bar graphs indicate that FcRn expression only in cm 4 and cm 5 of the intestine from FcRn^+/+^ strains was statistically significantly greater than in the FcRn^−/−^ tissues.

Aiming to study enterocytes expressing maximal concentrations of FcRn, we assessed by IB the expression of FcRn along the length of neonatal mouse duodenum and jejunum using a polyclonal rabbit anti-rat FcRn antibody that cross reacts with mouse FcRn [33]. This antibody recognized one or two bands of ∼50 kDa in lysates of neonatal intestine from FcRn^+/+^ but not FcRn^−/−^ pups (Fig. 2A). The signal was weak yet visible in cm 1, representing duodenum and perhaps the beginning of jejunum; the signal then increased along the length towards jejunum peaking at cm 4–5, and decreased thereafter. The lysates from cm 1–3 gave only a single band but cm 4–7 gave an additional upper band of slower mobility. The presence of two bands for FcRn in IB has been described by others [20], [22], [39]–[40]. We quantified FcRn expression in cm 1 through 7 and found that cm 4 and 5 showed statistically significantly higher levels of FcRn in FcRn^+/+^ as compared to FcRn^−/−^ lysates (Fig. 2B). For subsequent IF studies only cm 5 showing maximum FcRn expression was used.

### FcRn expression in jejunal tissue sections

**Figure 3:**
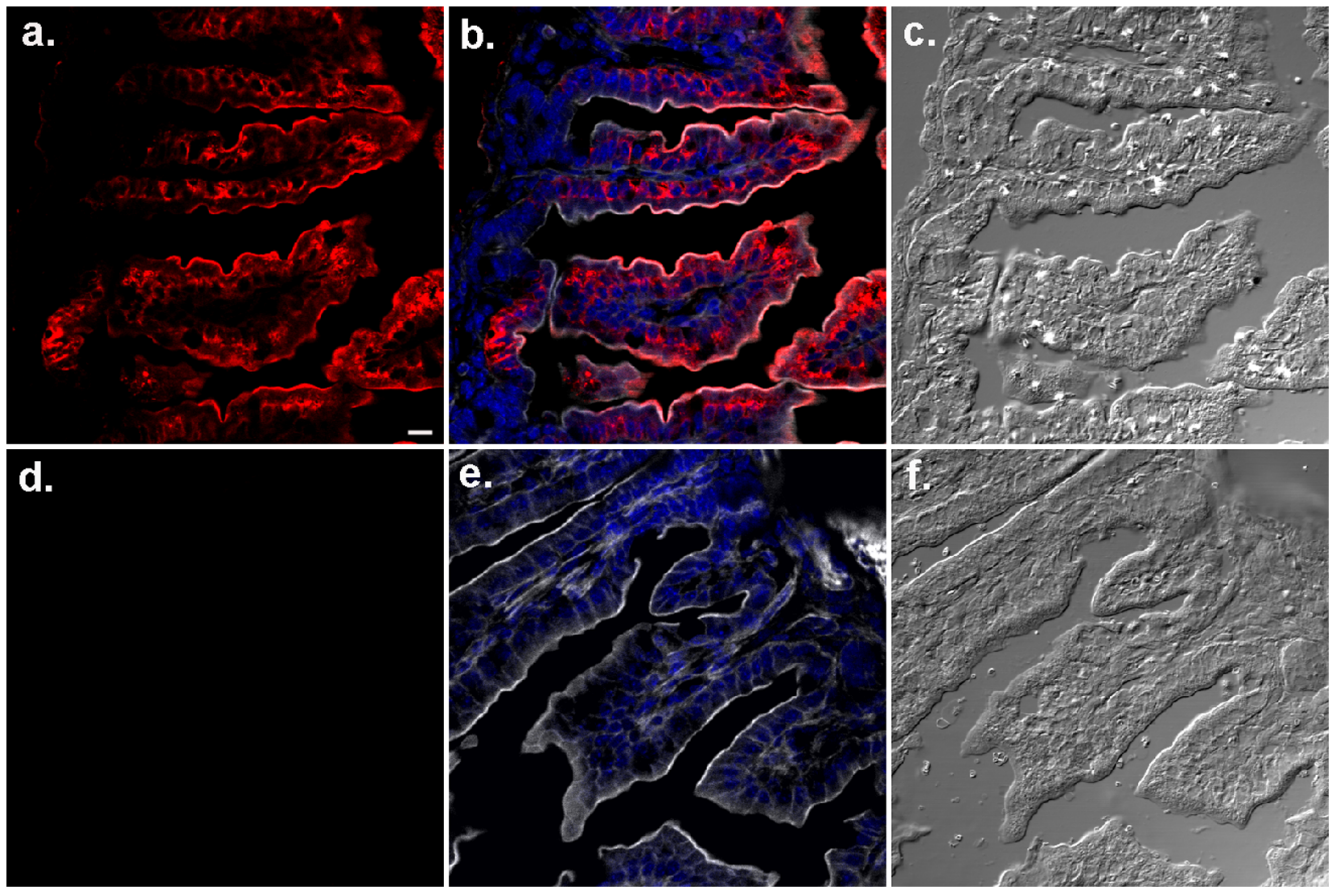
Specificity of hamster anti-mFcRn antibody in IF assay. Photomicrographs illustrating gut sections from FcRn^+/+^ (a, b, c) and FcRn^−/−^ (d, e, f) strain neonates labeled to visualize FcRn (red) with Armenian hamster anti-mouse FcRn antibody. The phalloidin (gray) and DAPI (blue) labeling were used to mark cell boundaries and nucleus, respectively, shown in b and e. For orientation DIC images in c and f are shown. The bar = 10 µm. See the complete lack of FcRn labeling in FcRn^−/−^ tissues (d) treated in parallel and in identical manner with FcRn^+/+^ tissues.

Sections of jejunum of FcRn^+/+^ and FcRn^−/−^ pups were labeled with hamster anti-mFcRn antibody. By IF microscopy we found that the distribution of FcRn was restricted to enterocytes of FcRn^+/+^ jejunum villi only (Fig. 3a and 3b). Goblet cells periodically interrupting enterocytes in epithelia were negative for FcRn expression. The core of the jejunum villi housing a variety of cells showed no FcRn expression. The FcRn^−/−^ tissue sections (Fig. 3d & 3e) showed complete absence of labeling affirming specificity of the antibody. The FcRn expression in FcRn^+/+^ gut was intense in the top three-quarters of the villus length, and diminished slowly to become completely absent at the base. The cells in the crypt at the base of the villi devoid of FcRn likely represent undifferentiated enterocytes.

### FcRn expression in neonatal jejunal enterocytes

**Figure 4:**
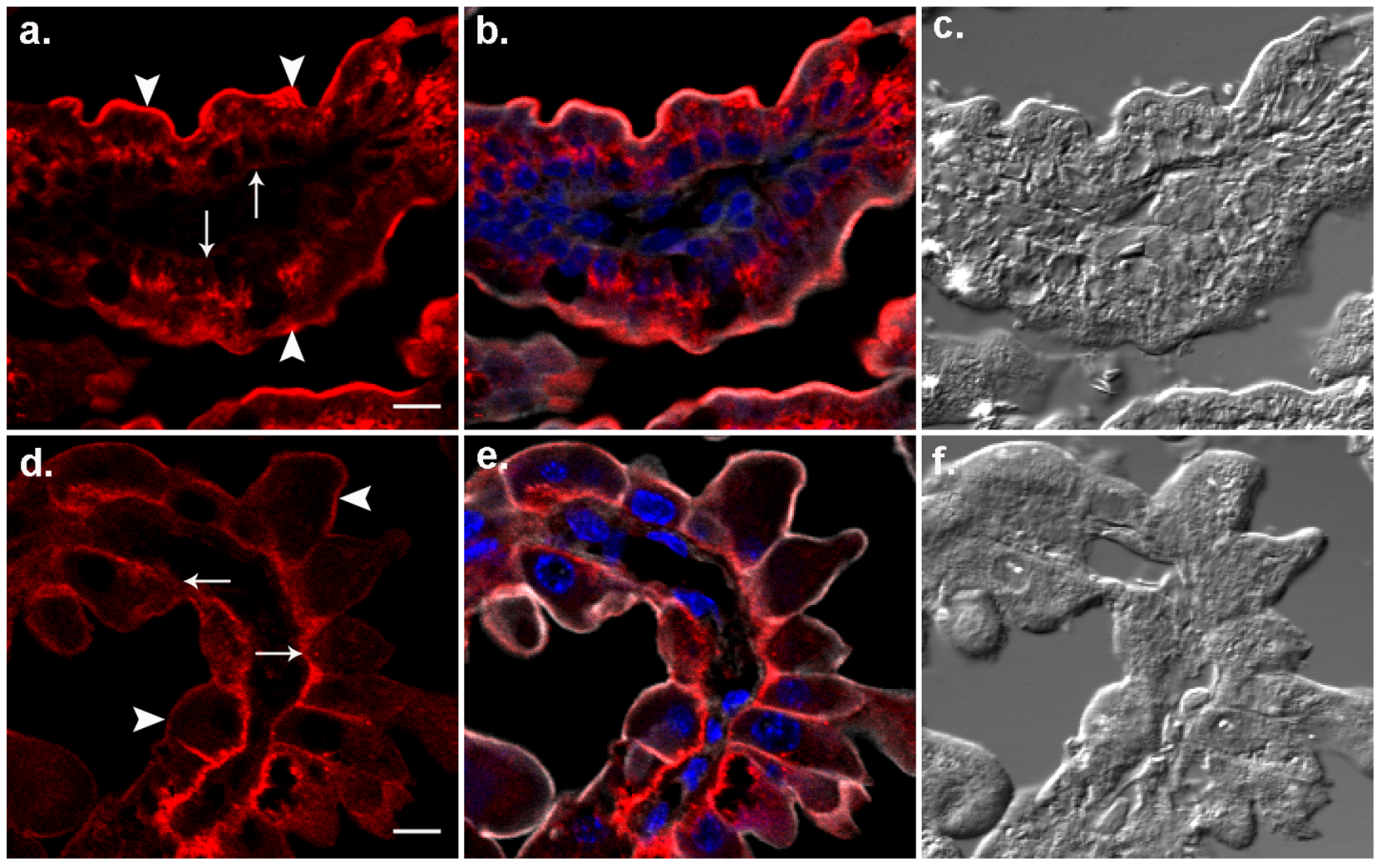
FcRn (red) distribution patterns in gut enterocytes and YS endoderm. Photomicrographs illustrating gut sections from FcRn^+/+^ neonates (a, b, c) and YS from FcRn^+/+^ tissues (d, e, f) are shown. The phalloidin (gray) and DAPI (blue) labeling were used to mark the cell boundaries and nucleus, respectively. For orientation DIC images in c and f are shown. Solid arrows point basilar and arrow heads point apical margins of the cells. The bar = 10 µm.

Below the brush border of enterocytes within the cytoplasm was a band-like terminal web composed of small vesicles and tubules making an interconnected network that labeled strongly with actin filament-binding phalloidin [41]. The FcRn signal in the apical margins of enterocytes coincided with the phalloidin signal (Fig. 4a). Therefore, we localize FcRn to the terminal web or the plasma membrane or both; the precise sites we cannot discern at this resolution. The signal was weak or nil at the basal margins. The interior of the cells showed FcRn signals as blobs and tubular structures in the supranuclear areas.

### FcRn was distributed differently in endodermal cells and enterocytes

To compare sub-cellular localization of FcRn between these two cell-types by confocal IF microscopy we labeled sections of both YS and jejunum from the FcRn^+/+^ strain with hamster anti-mFcRn antibody and phalloidin. Visual analyses of IF images of both endodermal cells and enterocytes showed abundant receptor expressed at the cell margins defined by the phalloidin signal. The FcRn signal associated with the cell margin was strong at apical (arrow heads) but weak at basilar (solid arrows) portions in the enterocyte (Fig. 4a), while the signal was evenly distributed on the cell margin of the endodermal cell (Fig. 4d). The FcRn signal in the cytoplasm, interior to the phalloidin signal, appeared in the enterocyte as supranuclear blobs and tubules spread between the nucleus and the apical margin whereas in endodermal cells the cytoplasmic FcRn signal was visible as tiny blobs in the basal parts of the cell. These differences may relate to the relative location and abundance of Golgi bodies as noted in the literature [16], [37].

To quantify these differences in receptor expression, we tabulated pixel intensities of receptor signals and calculated P/I, P_A_/P_B_ and I_A_/I_B_ ratios for both endodermal cells and enterocytes (Table 1). Quantifications of P/I ratios indicated that in the endoderm twice as much FcRn was coincident with the cell margin as with the cytoplasm whereas in the enterocytes the distribution between cell margin and cytoplasm was equal. The P_A_/P_B_ ratios of 300 enterocytes were almost 3 times greater than 300 endoderm cells indicating that in enterocytes FcRn is 3 times more intense in apical margin than the basal margin whereas endoderm showed relatively equal receptor distribution in both margins. The I_A_/I_B_ ratio of 300 enterocytes was twice greater than 300 endodermal cells supporting our visual impressions.

### IgG uptake by enterocytes does not require FcRn

**Figure 5:**
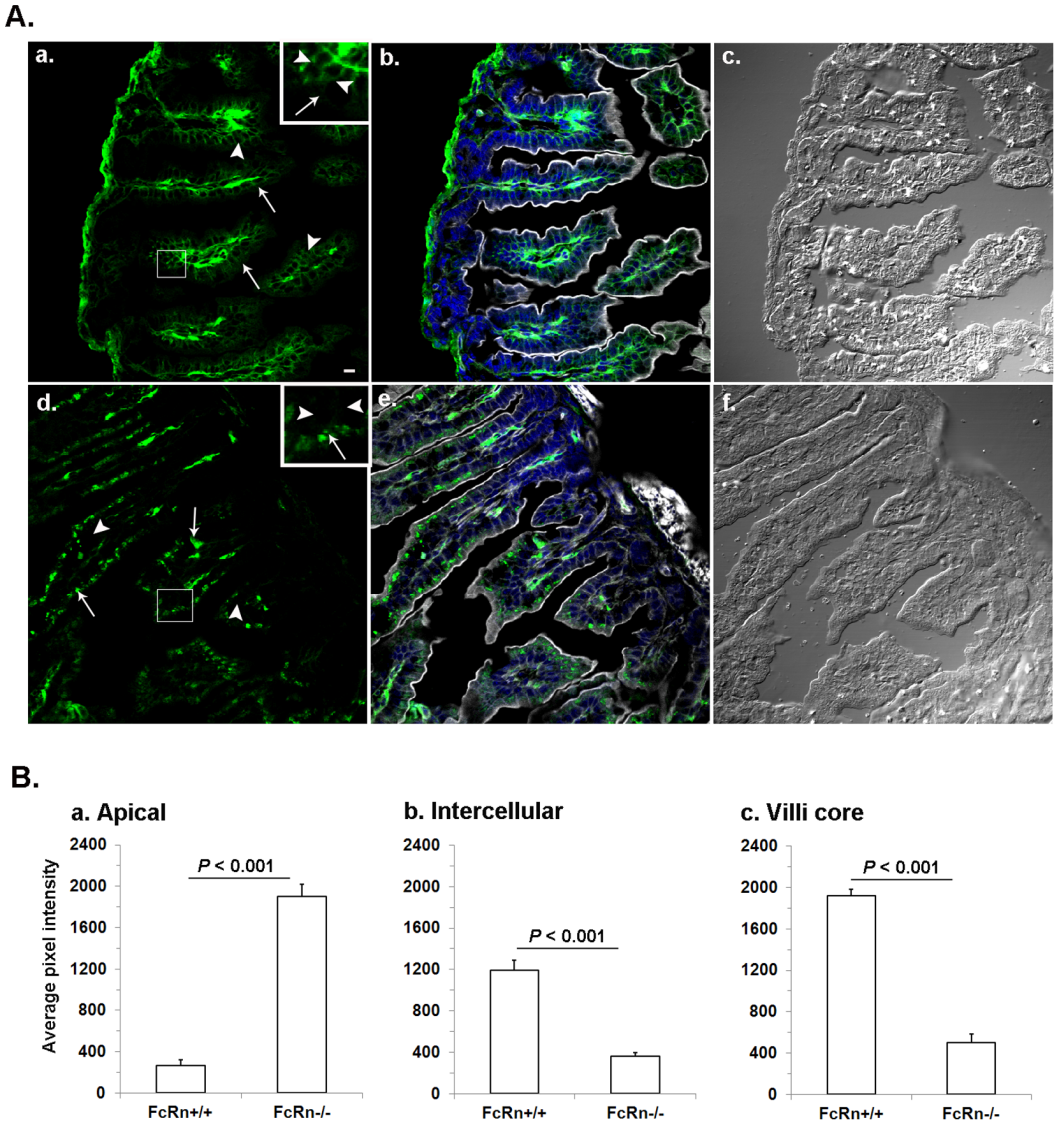
A comparison of IgG distribution between FcRn^+/+^ and FcRn^−/−^ neonatal gut. **A.** Photomicrographs illustrating gut sections from FcRn^+/+^ (a, b, c) and FcRn^−/−^ (d, e, f) neonates labeled to visualize IgG (green) with goat anti-mouse IgG heavy chain Fc antibody. The phalloidin (gray) and DAPI (blue) labeling were used to mark cell boundaries and nucleus, respectively, as shown in b and e. For orientation DIC images in c and f are shown. The bar = 10 µm. The insets of a and b contain a higher magnified view of an outlined area of FcRn^+/+^ and FcRn^−/−^ neonatal gut villi, respectively. The arrow heads point to the dense green labeling in intercellular areas of FcRn^+/+^ enterocytes and lack of it in FcRn^−/−^. The presence or lack, respectively, of dense green blobs in FcRn^−/−^ and FcRn^+/+^ apical areas is marked by a solid arrow. **B.** Quantitative analyses of IgG in apical, intercellular and core villi areas are shown. Fluorescence images such as shown in panel A (a and c) were collected on a confocal microscope. The images were quantified, averaged for 300 cells, and the average intensity plus or minus standard deviations was plotted for each strain (n = 3 FcRn^+/+^ and 3 FcRn^−/−^ mice). **a.** The FcRn^−/−^ apical area filled with green structures measured to be ∼7 times more intense than FcRn^+/+^ areas. **b.** The intercellular IgG in FcRn^+/+^ is ∼3 times more intense than FcRn^−/−^ intercellular areas. **c.** IgG in the FcRn^+/+^ jejunum villi core is ∼4 times more intense than the FcRn^−/−^ villi core areas.

Brambell's hypothesis that FcRn specificity was determined intracellularly in enterocytes would be compatible with an abundance of IgG within enterocytes of the FcRn^−/−^ strain. Indeed, we found abundant IgG within the enterocytes of the FcRn^−/−^ strain, appearing as intense blobs in the apical cytoplasm (Fig. 5Ad, solid arrow). In contrast, such blobs were not present in the apical cytoplasm of FcRn^+/+^ enterocytes; rather, only a weak amorphous receptor signal was evident (Fig. 5Aa, solid arrow). Further, our visual analysis revealed two other sites where IgG distribution was remarkably different between FcRn^+/+^ and FcRn^−/−^ gut. First, the intercellular areas in FcRn^+/+^ gut were distinctively rich in IgG whereas this area in FcRn^−/−^ gut was virtually devoid of IgG (Fig. 5Aa and 5Ad, arrow heads). Second, the villi cores of FcRn^+/+^ gut showed plentiful IgG while the villi cores of FcRn^−/−^ gut were IgG poor.

We quantified these visual impressions by collecting the average IgG intensities in apical cytoplasm of enterocytes, intercellular areas, and cores of the villi (Fig. 5Aa and 5Ad). Our analysis indicated that the pixel intensities of the IgG blobs in FcRn^−/−^ were 7 times more intense than the amorphous signal present in FcRn^+/+^ (Fig. 5Ba). The intercellular areas of FcRn^+/+^ had 3 times more IgG than FcRn^−/−^ (Fig. 5Bb). The FcRn^+/+^ cores of the villi had 4 times more IgG than the FcRn^−/−^ (Fig. 5Bc).

### IgG in the apical cytoplasmic area of endodermal cells was greater in FcRn^−/−^ than FcRn^+/+^

**Figure 6:**
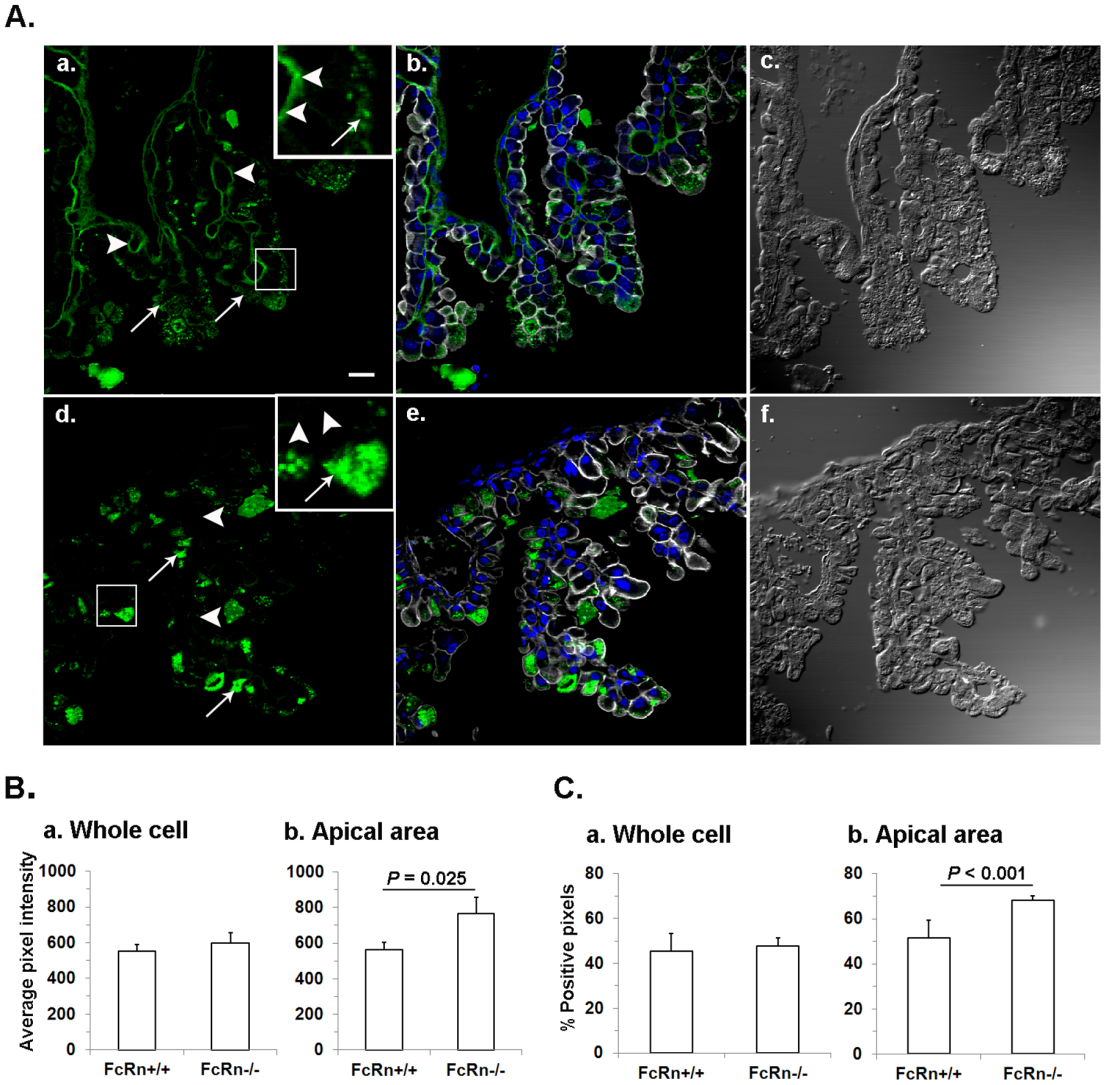
Comparison of distribution of IgG between FcRn^+/+^ and FcRn^−/−^ YS endoderm. **A.** Photomicrographs illustrating YS sections from FcRn^+/+^ (a, b, c) and FcRn^−/−^ (d, e, f) YS labeled to visualize IgG (green) as was done in Fig. 5. The phalloidin (gray) and DAPI (blue) labeling were used to mark cell boundaries and nucleus, respectively, shown in b and e. For orientation DIC images in c and f are shown. The solid arrows and arrow heads point to IgG in apical endoderm and mesenchyme, respectively. The bar = 10 µm. The inset of a and b, containing a higher magnified view of an outlined area of FcRn^+/+^ and FcRn^−/−^ yolk sac are shown. The solid arrows and arrow heads, respectively, point to IgG in apical and in mesenchyme areas in FcRn^+/+^. The lack of IgG in FcRn^−/−^ mesenchyme and the presence of dense blobs in apical areas are shown by arrow heads and solid arrows respectively. **B.** Quantitative comparison of IgG (average intensity) between FcRn^+/+^ and FcRn^−/−^ in whole cell and apical areas of ED. Fluorescence images such as shown in panel A (a and c) were collected on a confocal microscope. The images were quantified, averaged for 300 cells, and plotted as average intensity for each strain (n = 3 FcRn^+/+^ or 3 FcRn^−/−^ mice). **a.** The image shows that total IgG in FcRn^−/−^ is slightly higher but not statistically different than FcRn^+/+^ (*P* = 0.681). **b.** The image shows that IgG is more intense in FcRn^−/−^apical areas than in FcRn^+/+^. **C.** Number of pixels positive for IgG signal was quantified. Fluorescent images were converted to binary images and then used for quantifying the percent positive pixels. **a.** A difference could not be found in the percent of pixels positive for IgG in whole cell of FcRn^+/+^ and FcRn^−/−^ were almost equal (*P* = 0.554). **b.** Percent positive pixels in FcRn^−/−^ apical portion was significantly higher than in FcRn^+/+^.

Our earlier attempts to quantify the IgG signal between endodermal cells of FcRn^+/+^ and FcRn^−/−^ yielded no statistically significant differences [33]. Here, with a more refined reagent and a new approach, we again quantified the data and again found that total IgG intensity in the cytoplasm of endodermal cells of these two strains was similar (Fig. 6Ba). However, the distribution of intracellular IgG was different. In the apical portion of endodermal cell cytoplasm, IgG intensity was significantly greater (35%) in FcRn^−/−^ than in FcRn^+/+^ (Fig. 6Bb). Further, we compared the percentage, not the intensity, of positive pixels of the IgG signal. While the percentage of positive pixels of the IgG signal were comparable between endodermal cell cytoplasm of the two strains (Fig. 6Ca), the percentage alone of positive pixels in the apical regions was 17% greater in FcRn^−/−^ compared with FcRn^+/+^ cells (Fig. 6Cb).

## Discussion

Our principle aim was to affirm Brambell's hypothesis by demonstrating that cytoplasmic IgG is taken up nonspecifically in neonatal jejunal enterocytes of the FcRn^−/−^ strain. Indeed, in the cytoplasm of the FcRn^−/−^ enterocytes we found abundant IgG, appearing as numerous large blobs that were conspicuously absent from the cytoplasm of FcRn^+/+^ enterocytes where only an amorphous minimal IgG signal was seen. Remarkably, the IgG signal in FcRn^−/−^ cytoplasm was 7-fold that in FcRn^+/+^ cytoplasm. Our findings are compatible with Brambell's contention that receptor specificity for ligand is conferred intracellularly and not necessarily at the apical plasma membrane as Rodewald and Waldmann challenged.

Further, we call attention to the differences in density of the IgG signal in the extra-enterocyte areas of the jejunum between FcRn^+/+^ and FcRn^−/−^ strains. The intercellular and villi core areas of the gut show abundant IgG in the FcRn^+/+^ but very little in the FcRn^−/−^ strain. Thus, while IgG can enter the enterocyte without FcRn, it requires FcRn to be exocytosed at the basolateral side of the cell. The situation is very similar to what we earlier demonstrated for the YS [33] and confirm herein, and both situations are compatible with Brambell's hypothesis.

In the YS endoderm, where the intracellular site of receptor specificity for ligand is not disputed, our experiments show equal amounts of IgG in the cytoplasm of FcRn^+/+^ and FcRn^−/−^ animals, quite unlike the situation in enterocytes. However, looking carefully at sub-cellular areas we find somewhat more (35%) IgG in the apical cytoplasm of endodermal cells of FcRn^−/−^ relative to FcRn^+/+^ animals. This difference at the apical region would suggest the presence of an IgG gradient over the length of the cell that is established by FcRn. Such a gradient indicates that FcRn serves to transport endocytosed IgG toward the basal surface of the cell, away from the apical surface, in linked short segments rather than in single cell-spanning segments that would serve to deplete FcRn^+/+^ cytoplasm of IgG to result in lower overall IgG in the cell. Such a theory would have to be refined with alternative experimental strategies.

Because these two cell types, endodermal cells and enterocytes, are both responsible for moving maternal IgG to the mouse just a few days apart in the chronology of development, we were attentive to the relative cellular distribution of FcRn in these two cells. In general, FcRn distribution in these two cells was similar, consistent with the similar function of both the cells and the receptor. In both cell types at least one-half of total cellular FcRn was coincident with the cell margin which we defined as colocalizing with the phalloidin signal. However, we readily acknowledge that we were technically unable to discern the plasma membrane, so we cannot say whether FcRn was plasma membrane-associated. It is our supposition that in our microscopic images the phalloidin signal, identifying polymerized actin, would be coincident with both the plasma membrane and a compartment of sub-membranous organelles. In both marginal and cytoplasmic sites the patterns of FcRn signal were distinguishable between the two cell types, but not in any manner that would suggest obvious and direct functional consequences, so it is enough to record these differences in our description of results.

Finding that our data presented here support Brambell's hypothesis of intracellular receptor specificity, we are challenged to reevaluate the strength of the original data of Rodewald and Waldmann that were said to counter that ligand specificity in the neonatal gut was conferred at the enterocyte plasma membrane [12], [14]. In retrospect, we find the evidence tenuous. While it seemed clear that the neonatal jejunal luminal contents were acidic (pH 6.3), appropriate for high affinity binding to a surface displayed FcRn [14], to our knowledge Rodewald's pH measurement with pH paper has never been repeated, so his conclusion should be considered tentative. Much weaker is the conclusion from evidence that IgG could be shown to bind to neonatal jejunum at 0°C at acid pH but not at physiological pH [14], for while this finding obviously defines FcRn binding, it could be that such binding is manifested by only a tiny fraction of the entire cellular complement of FcRn, the remainder being intracellular and out of the way of surface access. In fact, these workers eventually acknowledged that surface FcRn was a minor fraction of the cellular total [16]. Further, others showed that IgG absorption into the suckling circulation occurred regardless of gut luminal pH [15]. The data underpinning Waldmann's challenge are of two sorts. First, he showed that jejunal uptake, defined as the % of radiolabeled IgG placed in the gut lumen that could be measured in the gut wall after 45 minutes of 37°C incubation, was specific for IgG and that such uptake could be blocked by unlabeled IgG. Second, he showed that IgG was associated specifically with membranes prepared from purified microvilli. While he concluded that specificity was conferred at the plasma membrane, it would seem in light of today's insights that neither of these two experiments excludes the possibility that the first pinocytic step was nonspecific and that IgG then bound to an endosomal FcRn, with specific uptake at the plasma membrane constituting a small fraction of the total.

Brambell's model brilliantly unified two disparate functions of FcRn, describing a simple mechanism that explained its workings in all of its various sites in the body. An alternative explanation centering on a small fraction of FcRn expressed at the cell surface, both in enterocytes and in other cells as well, prompted doubt about the unifying hypothesis, a doubt that appears to continue to this day. A knock out mouse unavailable to the early workers has allowed us to determine unambiguously that FcRn is not required for uptake of IgG in enterocytes, establishing thus that receptor specificity may be conferred within the cell. Moreover, upon reassessment of the early publications, sufficient doubt now appears about the Rodewald-Waldmann challenge that we must still again conclude that Brambell's hypothesis is the best available [42].
